# Measuring Fluxes of Nitrous Oxide (N_2_O) From an Intensively Farmed Wasted Peatland Field in the UK Using the Eddy Covariance Method

**DOI:** 10.1111/gcb.70619

**Published:** 2025-11-21

**Authors:** Nicholas Cowan, Alex Cumming, Ross Morrison, Hannah Clilverd, Luke Palmer, Chris D. Evans

**Affiliations:** ^1^ UK Centre for Ecology and Hydrology Easter Bush Midlothian UK; ^2^ UK Centre for Ecology and Hydrology Crowmarsh Gifford Wallingford UK; ^3^ F.C. Palmer & Sons Ltd. Ely UK; ^4^ UK Centre for Ecology and Hydrology Environment Centre Wales Bangor UK

**Keywords:** agriculture, carbon, emission factor, greenhouse gas, nitrogen, soil moisture, tillage

## Abstract

While the number of studies quantifying emissions of the greenhouse gases (GHGs) carbon dioxide (CO_2_) and methane (CH_4_) from peatland farming has increased in recent years, high uncertainty regarding the magnitude and drivers of emissions of the powerful GHG nitrous oxide (N_2_O) from farmed peatland soils remains. This study used eddy covariance to measure fluxes of N_2_O over a 3‐year period from a commercial farm in the East Anglian Fens, in a rotationally cropped field with a 56 cm surface layer of peaty soil. Over the 41‐month measurement period, the average (±95% CI) monthly field‐scale emission was 0.50 ± 0.17 kg N_2_O‐N ha^−1^, which equates to approximately 6.0 ± 2.0 kg N_2_O‐N ha^−1^ year^−1^. Emissions of N_2_O at the field site were controlled by thresholds in both soil temperature (low fluxes below ~12°C) and volumetric water content (low fluxes below ~65%). Where these thresholds were simultaneously exceeded at any depth within the top metre of the soil profile, N_2_O emissions increased by an order of magnitude. Higher water level management in the summer months resulted in a significant increase in annual N_2_O emissions, estimated to be up to 10 kg N_2_O‐N ha^−1^ year^−1^ higher than in years when the water table was lower. Elevated emissions of N_2_O were largely controlled by environmental conditions (i.e., moisture and temperature). These conditions were in turn influenced by crop management, with higher emissions occurring when the field was cultivated for potatoes (compared to wheat and beans) which we attribute to a combination of higher water level management, overhead irrigation, relatively low crop nitrogen demand and solar heating of the exposed soil surface.

## Graphical abstract

Fluxes of nitrous oxide (N_2_O) were measured using the eddy covariance technique from a wasted peatland farm near the village of Stretham in Cambridgeshire, UK. Measurements lasted over 3 years, with a variety of crops, fertiliser applications and agricultural management practices occurring throughout. Where thresholds in soil moisture and soil temperature were simultaneously exceeded at any depth within the top metre of the soil profile, N_2_O emissions increased by an order of magnitude.

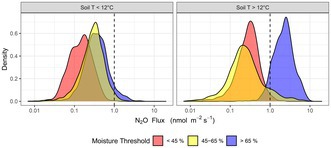

## 1 Introduction

The UK is one of the most peat‐rich countries in the world on an area basis, with peat occupying around 12% of the land area (Brown et al. 2024). Although the majority of this is upland blanket bog, around 15% of UK peatland occupies lowland areas, as either base‐rich fen peat or more acidic raised bogs. Historically, the cultivation of lowland peatlands was constrained by the difficulties of draining the naturally waterlogged soils, and land use was largely limited to summer grazing. However, increasingly mechanised large‐scale drainage from the 17th century onwards converted the vast majority of the UK's lowland peat area into highly productive farmland, supporting intensive arable, horticultural and grassland agriculture. While this conversion of lowland peat from wetland to farmland now contributes significantly to regional economic output and food production, it has also transformed ecosystems that previously acted as long‐term carbon dioxide (CO_2_) sinks and large carbon and nitrogen stores into substantial CO_2_ sources, as a result of the accelerated decomposition of peat under aerobic conditions (Lloyd et al. 2023).

A further consequence of this change has been widespread land subsidence, exceeding 3 m in some areas. Consequently, large areas of the East Anglian Fens now lie below sea level, and energy‐intensive pumped drainage is required to maintain current agricultural practices (Dawson et al. 2010). This subsidence results from a combination of the oxidation and compaction of drained peat and can continue for centuries. As this process of peat ‘wastage’ proceeds, the peat layer, which may originally have been up to several metres thick, gradually reduces in thickness whilst simultaneously increasing in bulk density. In areas that have been drained for centuries, or where the original peat layer was shallow, the remaining deposit no longer meets the threshold (40 cm in England and Wales, 50 cm in Scotland and Northern Ireland) to be described as true peat as they are often intermixed with mineral soil as a result of ploughing. These areas are typically referred to as either ‘wasted peat’ or ‘skirtland’. Due to their relatively high bulk density, wasted peats may still hold significant stocks of organic carbon (OC). However, if they remain under drainage and cultivation, this OC will continue to be oxidised to CO_2_, and the peat layer will continue to shrink.

In addition to subsidence and loss of agricultural output, peat wastage is contributing significantly to total greenhouse gas (GHG) emissions from the UK's land‐use sector. An assessment of data gathered by a network of flux measurement sites on lowland peatlands across England and Wales suggested that CO_2_ emissions from lowland peat under arable and horticulture were extremely high (estimated at 25 t CO_2_ ha^−1^ year^−1^), making them arguably the largest emissions hotspot within the UK's Agriculture, Forestry and Other Land Use (AFOLU) sector (Evans 2016). The assessment showed that high rates of CO_2_ emission were positively correlated with drainage depth and suggested (based on limited data) that N_2_O emissions made a substantial contribution to total GHG emissions at some fertilised sites (Taft et al. 2017). Emissions of N_2_O are a by‐product of the microbial processes of nitrification and denitrification, in which nitrogen in the form of ammonium (${\mathrm{NH}}_{4}^{+}$) and nitrate (${\mathrm{NO}}_{3}^{-}$) is converted to inert nitrogen gas (N_2_) as part of the natural nitrogen cycle (Davidson et al. 2000). As well as the application of nitrogen fertilisers to agricultural soils, organic matter in peatland soils also contains nitrogen, which is mineralised into microbially available compounds (e.g., ${\mathrm{NH}}_{4}^{+}$ and ${\mathrm{NO}}_{3}^{-}$) when peat wastage occurs. Our understanding of the fate of the nitrogen cycled in these soils is limited due to the lack of dedicated studies on farmed peatlands.

While significant investments have been made in establishing long‐term research infrastructure at many peatland sites in the UK, until recently there has been a lack of long‐term or continuous N_2_O measurements. Field N_2_O measurements at lowland peatlands in the UK (Evans 2016; Taft et al. 2017) as well as internationally (Regina et al. 2004; Maljanen et al. 2004; Drewer et al. 2010; Juszczak and Augustin 2013; Kandel et al. 2018; Minkkinen et al. 2020; Anthony and Silver 2021; Lin et al. 2022) have been limited to periodic manual chamber measurements at a limited number of sites. Where peatlands are left in their natural condition, fluxes of reported N_2_O are relatively small, due to acute ecosystem N limitation and a resulting lack of available N to permit nitrification and denitrification to occur, as well as waterlogged conditions that restrict nitrification and promote full denitrification (conversion of N_2_O to N_2_). However, changes associated with agricultural land use, including N enrichment, soil aeration and disturbance, can lead to high emissions (Pihlatie et al. 2010; Prananto et al. 2020; Lin et al. 2022; Schaller et al. 2022). Where N_2_O emissions are high in soils, uncertainties in spatial and temporal interpolation scale exponentially (Levy et al. 2017) and flux data collected from chamber measurements are limited in how well they can capture and explain the extreme variations in emissions like those observed from agricultural peatlands (Anthony and Silver 2021). For this reason, it would be advantageous to measure N_2_O emissions from peatlands using the eddy covariance technique which is capable of continuous measurement of emissions with large spatial coverage (> 100 m^2^).

Continuous measurement of N_2_O flux using eddy covariance is technically challenging, requiring expensive instrumentation and uninterrupted mains electricity (Nemitz et al. 2018). To date, very few N_2_O eddy covariance sites have been setup for long‐term flux monitoring in the UK, with only one long‐term study (reporting up to 4 years) of flux measurements from a mineral soil agricultural field in Scotland (Cowan et al. 2020). The eddy covariance method has been used at very few agricultural peatland sites around the world to measure N_2_O fluxes, but results from these studies consistently agree that these soils can be a large source of emissions (e.g., Kroon et al. 2010; Gerin et al. 2023). This study aims to increase our understanding of N_2_O emissions from wasted peatland soils under intensive agricultural management in the UK. Our study intends to better quantify the magnitude of emissions at field scale, and to identify potential environmental or management‐related drivers that significantly alter N_2_O flux dynamics.

## 2 Method

### 2.1 Field Site and Management

**FIGURE 1:**
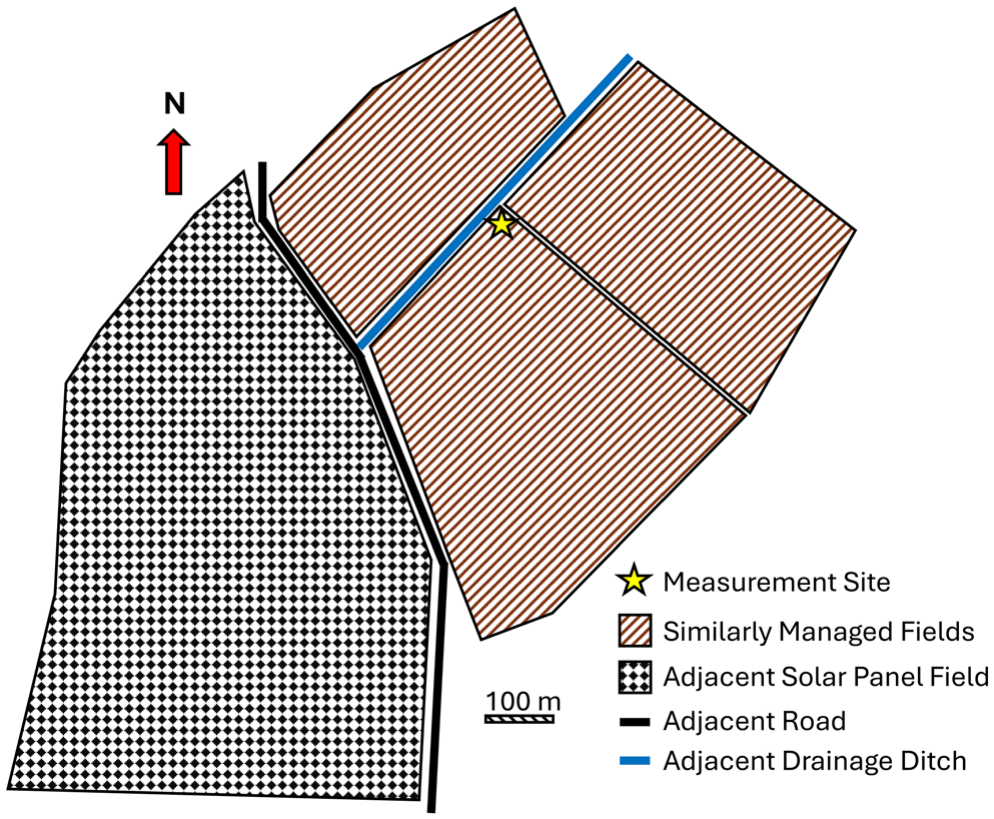
Location of eddy covariance flux tower at the centre of three similarly managed arable fields at Stowbridge Farm.

Measurements were carried out at Stowbridge Farm (F.C. Palmer & Sons Ltd.) near the village of Stretham in Cambridgeshire, UK. The measurement site is close to the Stretham Old Engine, the last of the steam‐powered pumps that were used to drain the Fenland during the nineteenth and early twentieth centuries. The farm is under conventional agricultural management, producing cereals (e.g., wheat), vegetables (beet, potatoes, etc.) and sports turf. The flux tower is located at a central location between three fields with a combined area of 36 ha (Figure 1). These fields are managed together as a single unit. The wind direction at the site is predominantly from the southwest (typical of the UK), the mean annual temperature is 10.7°C and total annual rainfall is 708 mm (UK Met. Office). The soil adjacent to the flux tower is classified as an earthy eutro‐amorphous peat soil of the Adventurers' series, with a 0.56 m layer of peaty loam over 0.12 m of humified peat and a further 0.09 m of humose silt loam, identified as buried topsoil. Underlying mineral soils are loamy drift over greensand. The field contains a series of parallel land drains at 20 m spacing which facilitate water movement from the field into surrounding ditches, in turn feeding into the regional drainage network.

**FIGURE 2:**
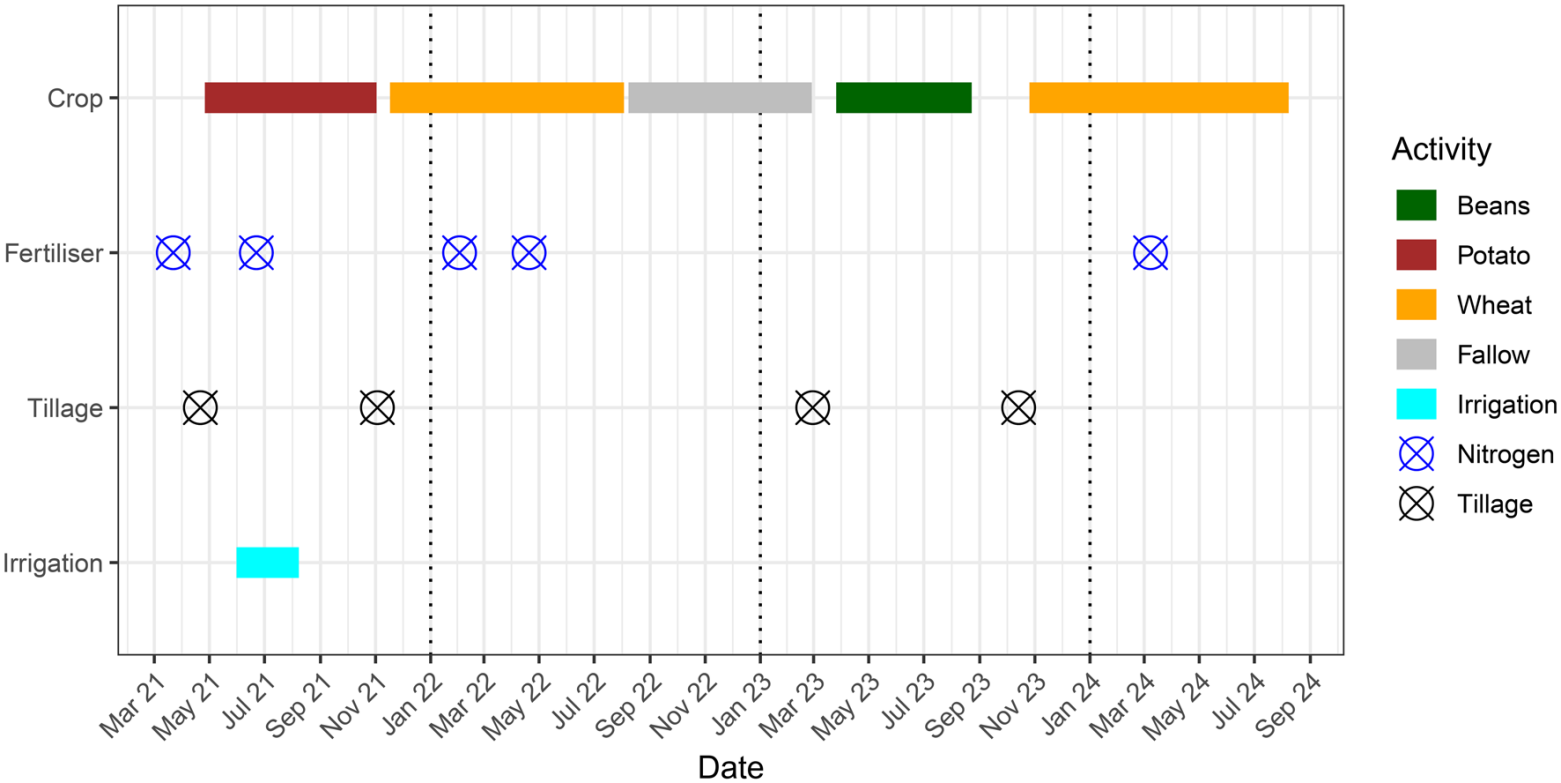
Field management activity at the N_2_O eddy covariance site at Stowbridge Farm March 2021 to October 2024. Crops present in the field, and dates of nitrogen fertiliser application, tillage events and irrigation activities are highlighted.

Measurements of N_2_O flux began shortly after the planting of a potato crop on 27 April 2021. Potatoes remained in the fields until harvest on 3 November 2021. The water table was lowered at the end of 2021 to below land drain depth. Wheat ( *Triticum aestivum* ) was drilled immediately after the potato harvest and harvested on 3 August 2022. Fields were left uncultivated during the intercrop periods and experienced natural colonisation by volunteer weed species until 26 March 2023 when green beans ( *Phaseolus vulgaris* ) were planted, which were then harvested on 23 August 2023. After a second short intercrop period, wheat was planted on 17 November 2023 and remained in the fields until harvest on 8 August 2024 (Figure 2). Mechanical agitation of the soil occurred during the harvest and subsequent tillage of the fields before and after each crop was harvested. Potato crops were planted on shallow ridges and intermittently irrigated using nearby ditch water for the period of 31 May 2021 to 8 August 2021 using an overhead sprinkler system. Mineral nitrogen fertiliser was applied prior to the planting of the potato crop (40 kg N ha^−1^ on 22 March 2021) and then on 22 June 2021 (64 kg N ha^−1^). Nitrogen fertiliser (52 kg N ha^−1^) was applied twice to the wheat crop on 2 February 2022 and 21 April 2022. No nitrogen fertiliser was applied to the bean crop. Nitrogen fertiliser (39 kg N ha^−1^) was applied to the wheat crop on 8 March 2024 (Figure 2).

### 2.2 Meteorological Measurements

A suite of supporting instrumentation was utilised to provide half‐hourly resolution meteorology which was logged on a Campbell Scientific CR3000 datalogger (CSI, USA). Air temperature and relative humidity were measured using HMP155A (Vaisala, Finland) while a nine‐point profile of soil temperature and volumetric water content was measured using a SoilVUE10 soil profiler (CSI, USA) at 0.05, 0.1, 0.2, 0.3, 0.4, 0.5, 0.6, 0.75 and 1 m. A tipping bucket rain gauge SBS500 (Environmental Measurements Limited, UK) was used to record precipitation at a height of 1 m, and water table depth was recorded using a CS451 pressure transducer (CSI) which was installed in a dipwell below the water level.

### 2.3 Eddy Covariance Flux Measurements

A mast was setup (8 May 2021) to measure continuous fluxes of nitrous oxide (N_2_O) using the eddy covariance technique. A continuous wave quantum cascade laser (QCL) absorption spectrometer gas analyser (CW‐QC‐TILDAS‐76‐CS, Aerodyne Research Inc., Billerica, MA, USA) was housed within an air‐conditioned container within a mobile trailer unit that was connected to a mains power source. A sonic anemometer (Gill Windmaster Pro, Hampshire UK) was fitted to the mast, at a height of 3 m which measured fluctuations in 3‐D wind components at a frequency of 20 Hz. A 20 m length of 3/8″ ID Dekabon tubing was fitted between the anemometer and the QCL instrument as an inlet, which drew air at a flow rate of approximately 14 L min^−1^ via a vacuum pump (Triscroll 600, Agilent Technologies, US). The QCL instrument measured gas concentrations of N_2_O, carbon dioxide (CO_2_) and water (H_2_O) at a rate of 10 Hz. Data from the sonic anemometer was logged on a data logger (CR1000, CSL, USA), while data from the QCL was logged internally. A GPS clock was used to ensure logged data remained synchronised throughout the campaign and could be merged accurately afterwards at 20 Hz (with concentration data matched to the nearest decimal second).

Fluxes were calculated over 30‐min intervals using the EddyPro software (Version 7.0.9, Li‐COR, Lincoln, NE, USA), based on the covariance between gas concentration (χ) and vertical wind speed (ω) ($F=\bar{\chi \omega }$). The time lag between the inlet and the QCL instrument was checked monthly using the maximisation of covariance between the vertical wind component and CO_2_ for which the signal was consistently stronger than N_2_O. In the flux calculation processing, we applied double coordinate rotation (vertical and crosswind), spike removal, block averaging and outlier removal of artefact measurements. The high and low frequency cospectral attenuation of the system was corrected using the analytical method of Moncrieff et al. (1997). Corrections for density fluctuations in response to changes in temperature and humidity were applied on a half‐hourly basis using the method of Ibrom et al. (2007). The quality control scheme of Mauder et al. (2013) was used to remove poor quality flux measurements (Category 2). Data were also rejected on the basis of extreme outliers and friction velocity (u*) values < 0.1 m s^−1^.

Cumulative emissions of N_2_O were estimated using a simplified general additive model (GAM). This accounted for temporal patterns at a range of time scales and nonlinear responses to environmental variables, implemented using the mgcv package in the R software (Wood 2017). The GAM was fitted to the flux data, using only temporal variables (day of year, month, year) and the maximum volumetric water content (VWC) of the soil profile and the temperature of the soil at the corresponding depth during a half‐hour period (e.g., for the same period as the flux was averaged). Where power cuts prevented the collection of data for modeling purposes, linear interpolation was used.

## 3 Results

### 3.1 Meteorological and Soil Conditions

**FIGURE 3:**
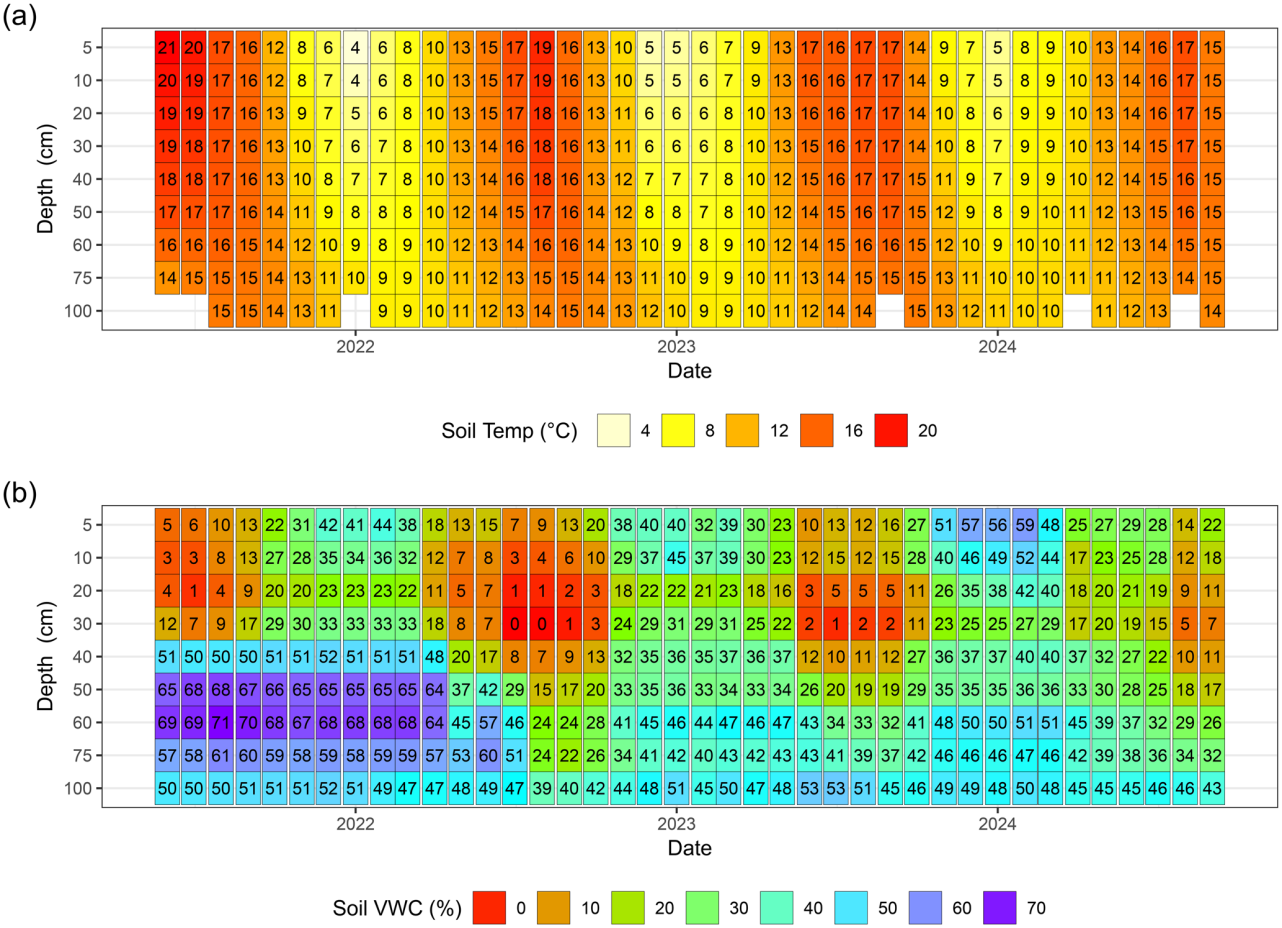
Monthly mean (a) soil temperature and (b) volumetric water content (VWC) of the soil profile at the Stowbridge Farm flux tower site.

Environmental conditions at the site varied over the 3‐year period, with multiple deviations from what would be considered climatically normal. For the majority of the study period, air and surface (5 cm) soil temperatures were closely coupled, following a regular annual oscillation with monthly mean soil temperatures near 5°C during the winter and reaching near 20°C during the peak of summer (Figure 3). However, air and surface soil temperatures partly decoupled during 2021, when the potato crop was present and was shading the soil (and the site was also periodically irrigated). During this period, monthly mean air temperature peaked in July at 16.9°C (max 27.3°C) whereas soil temperature rose earlier and higher with monthly means of 19.8°C in both June and July, and a maximum temperature of max 36.6°C (Figure S2). Conversely, in 2022, when the wheat crop was present, growing season air and surface soil temperatures were similar, with mean monthly air temperature peaking at 18.6°C in July, and surface soil temperatures peaking at 18.9°C in August. During a heatwave in July 2022, air temperatures in the region reached their highest levels ever recorded, peaking on our site at 37.4°C on 19 July 2022. Despite this, surface soil temperatures only reached 22.0°C during the same period. In 2023 (bean crop) conditions were generally cooler, but a similar pattern was observed whereby peak surface soil temperatures were about 10°C lower than peak air temperatures.

Cumulative annual rainfall averages varied widely across the measurement period, with 467 mm recorded in 2022 and 877 mm recorded in 2023 (a difference of 87%). In September to October of 2023, 345 mm of rain fell in the field, equivalent to 74% of the total rainfall for 2022 (Figure S1). Due to the drainage network around the field and active water management at the farm scale, increases to the soil moisture content of the soil were driven by rainfall and hydrological management. A repeating pattern of dry and wet conditions in the seasonal cycle can be observed in the top 40 cm of the soil, varying from very dry conditions (< 10% VWC) in summer to relatively high VWC content (over 50%) in winter. Soil moisture in the growing season of 2022 was particularly low due to the very low rainfall in 2022 and extended agricultural drought conditions, and high in the winter of 23/24 due to the very high concentrated rainfall. In 2021, the drainage ditches were maintained at relatively high levels to aid with the irrigation needs of the potato crop in the fields. After the drainage levels were lowered, the soil moisture in the deeper layers of the soil (> 40 cm) fell significantly from > 60% VWC to < 50%, and did not reach these same moisture contents for the rest of the duration of measurements (Figure 3).

### 3.2 N_2_O Flux

**FIGURE 4:**
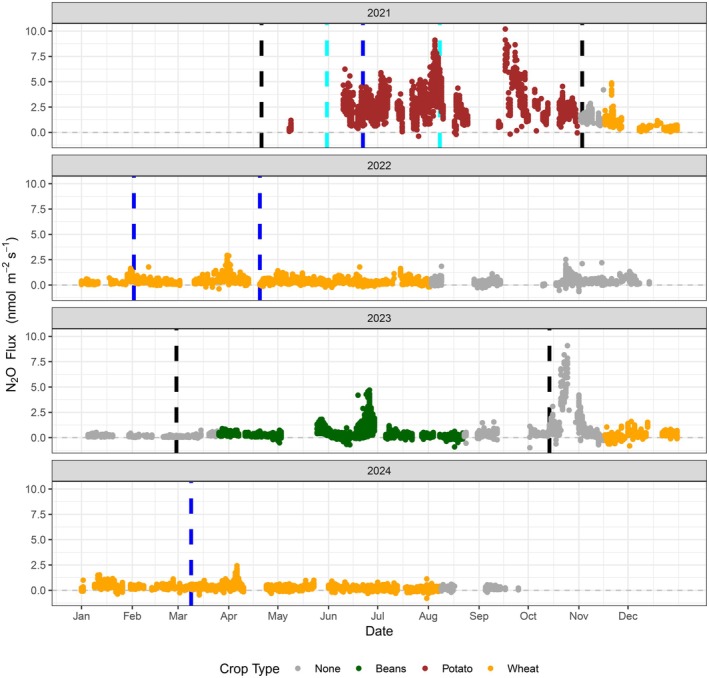
Nitrous oxide (N_2_O) fluxes measured using the eddy covariance method at the Stowbridge Farm flux tower site. Management activities are denoted with vertical lines (blue for nitrogen application and black for tillage events). Irrigation of the potato crop lasted from the 31 May 2021 to 8 August 2021 (cyan).

Fluxes of N_2_O varied exponentially on a temporal scale, ranging from −0.9 to 10.2 nmol m^−1^ s^−1^. The combined flux data coverage of the period in which measurements passed quality controls was 48% (Figure 4); however, several periods of measurement data are missing due to power outages and instrument failures. The wind was predominantly south‐westerly, accounting for 43% of wind conditions (Figure S3a). Peak contributions to the flux footprint at the site were typically 30 to 60 m from the tower position (Figure S3b). The median of all measured fluxes at the field was 0.31 nmol m^−2^ s^−1^, and the majority of measured fluxes were low (71% of measurements < 0.5 nmol m^−2^ s^−1^), with sporadic periods of considerably higher emissions. Key emission events that stand out from background flux conditions at the site included (i) large, sustained emissions throughout the potato growing season in 2021, (ii) a large peak in emissions midway through the bean growing season in 2023 and (iii) a large, sustained peak in emissions after tillage of the bean crop into the soil, also in 2023 (Figure 4). There was no immediate N_2_O emission response (i.e., within ~4 weeks) after the application of nitrogen fertilisers for any of the application events.

**FIGURE 5:**
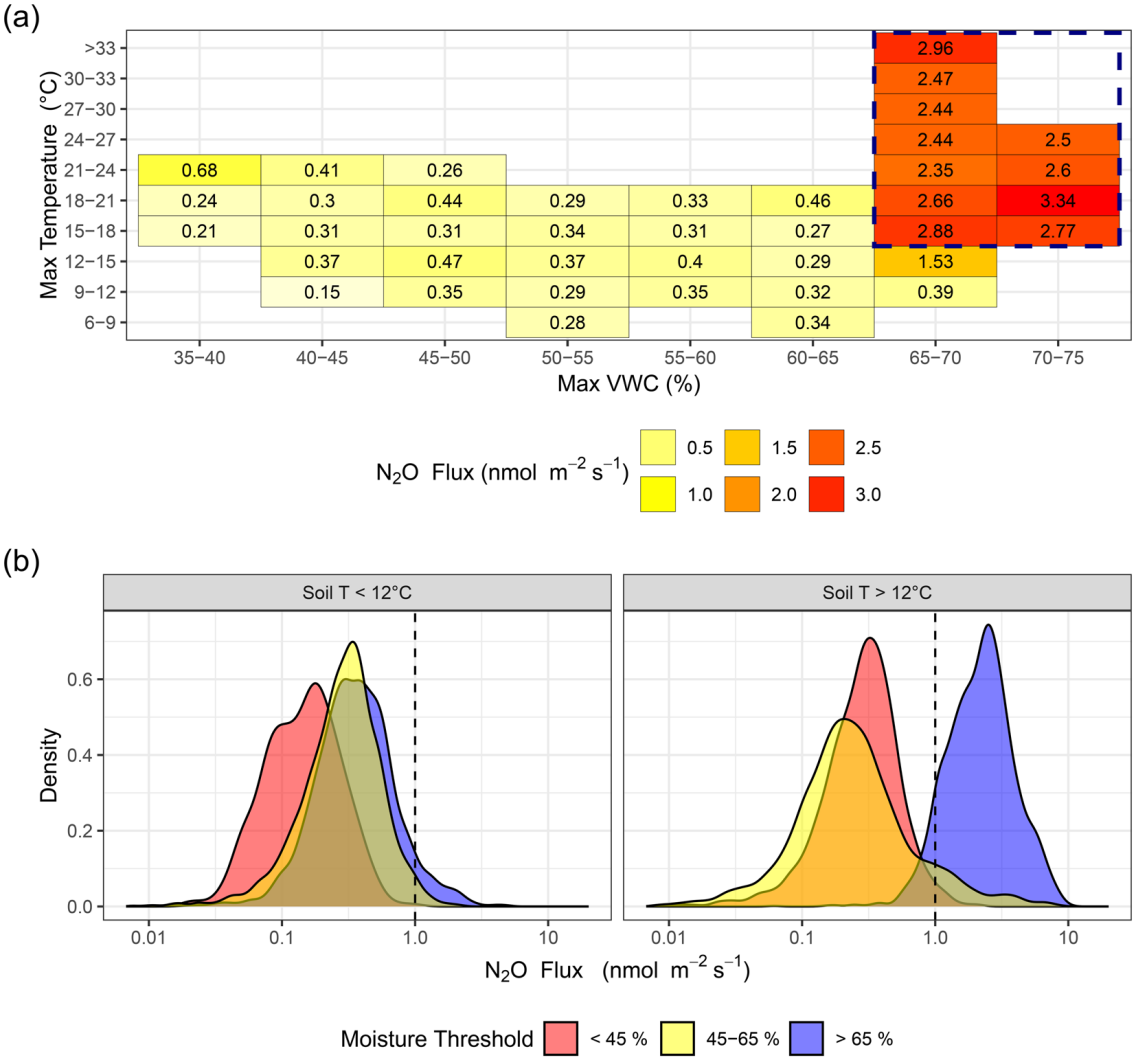
(a) Mean N_2_O flux data are presented, binned by maximum soil temperature and maximum VWC content (at any depth in the top 1 m of soil). Fluxes that pass the threshold indicators (temperature > 12°C and VWC > 65%) are outlined (blue dashed box). (b) Distribution of N_2_O flux data on a log‐scale, binned into conditions where soil temperature (at the depth of maximum VWC content) is above or below a 12°C threshold, and VWC (at any depth in top 1 m of soil) is below 45%, between 45% and 65% or above 65%.

Farm activities such as tillage resulted in inconsistent emissions of N_2_O. Emissions of N_2_O from the field site do not correlate directly with any of the individual environmental parameters; however, clear trends are still present in the data. While the majority of N_2_O emissions remained below a threshold of 0.5 nmol m^−2^ s^−1^ during all temperature conditions, there was a significant increase in the range of observed N_2_O emissions when soil temperature at any depth down to 1 m exceeds a threshold of 12°C (Figure S4). Similarly, the data suggest a threshold VWC of approximately 65% at any given depth up to 1 m, above which high N_2_O emissions may occur (Figure 5). When both temperature and moisture thresholds were met and coincided at the same depth, emissions of N_2_O increased exponentially with temperature. The dual threshold of 12°C and 65% VWC results in a shift from conditions where fluxes are almost all lower than 1 nmol m^−2^ s^−1^ to > 1 nmol m^−2^ s^−1^ (Figure 5); however, the flux data follows a log‐normal distribution, resulting in an exponentially large spread of measured flux when conditions pass the threshold (as observed during the potato crop of 2021). Fluxes of N_2_O were found to correlate with measured VWC in the soil, but not directly with water table depth (Figure S5). Fluxes were highest in 2021 when the summer water table was highest; however, periods of high VWC persisted in the top 1 m of soil even when the water table was lower (> 1 m depth).

**FIGURE 6:**
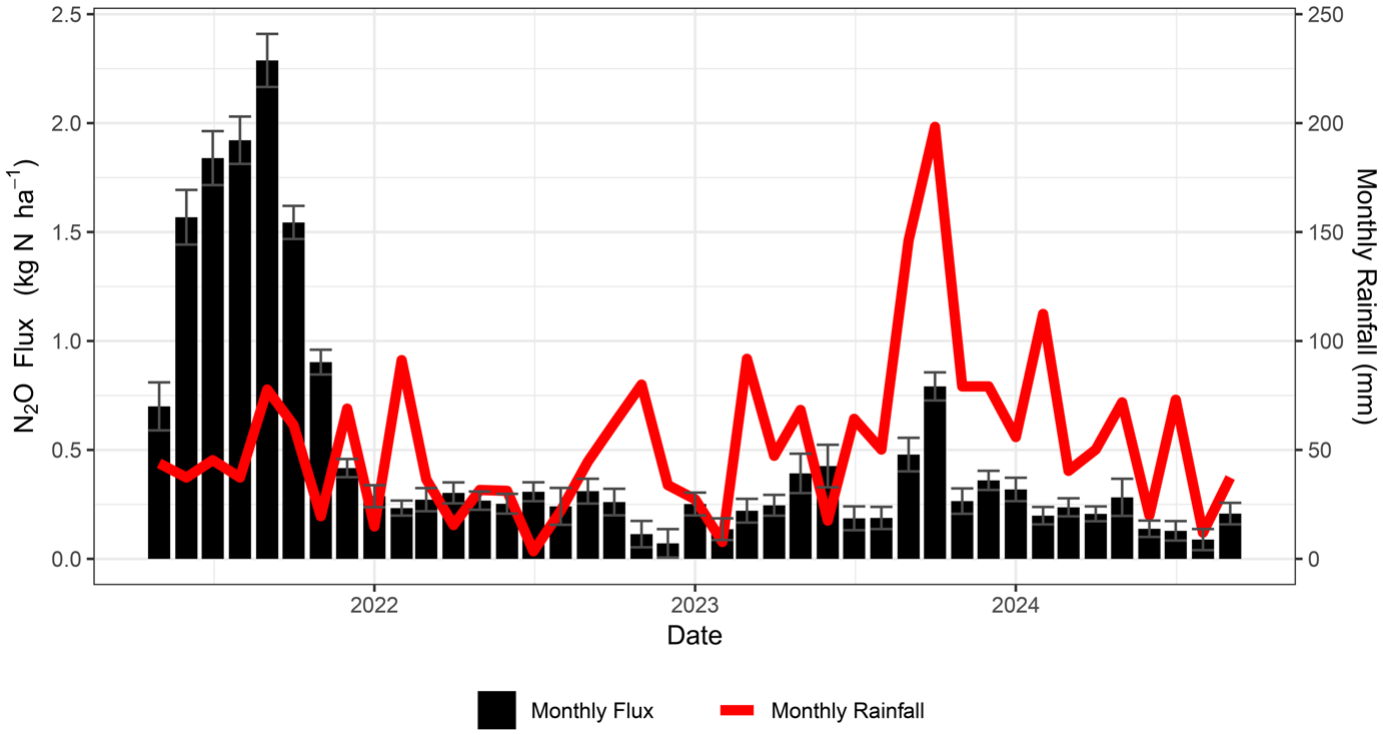
Cumulative monthly N_2_O flux is shown for the period over which measurements were made (May 2021 to September 2024), with monthly rainfall included for comparison. Error bars represent 95% confidence intervals.

Combined cumulative emissions for the measurement period (~3 years) were 20.4 kg N_2_O‐N ha^−1^ (32.1 kg N_2_O ha^−1^, or 8.51 tCO_2eq_ ha^−1^ (GWP of 265 over a 100‐year period)). However, 11.8 kg N_2_O‐N ha^−1^ (58%) of these emissions were attributed to 8 months of measurements in 2021 (Figure 6b). Yearly cumulative emissions of 2.90 and 3.90 kg N_2_O‐N ha^−1^ were estimated for the full duration of 2022 and 2023, with an additional 1.84 kg N_2_O‐N ha^−1^ released in the first 9 months of 2024. The average monthly emission from the field is 0.50 kg N_2_O‐N ha^−1^, ranging from 0.11 to 2.07 kg N_2_O‐N ha^−1^ over the measurement period (log‐normal distribution). Over all 41 months, the average (with 95% CIs) monthly emission is 0.50 ± 0.17 kg N_2_O‐N ha^−1^, which equates to approximately 6.0 ± 2.0 kg N_2_O‐N ha^−1^. Excluding data from 2021, the monthly emission from the fields is 0.26 ± 0.04 kg N_2_O‐N ha^−1^ year^−1^, which equates to approximately 3.1 ± 0.5 kg N_2_O‐N ha^−1^.

## 4 Discussion

Few studies have carried out long‐term measurements of N_2_O using the eddy covariance method, and those that have predominantly focus on grasslands and mineral soils (e.g., Merbold et al. 2014; Cowan et al. 2020; Murphy et al. 2022). This study is the first to provide long‐term measurements from an intensively farmed peatland in the UK and provides valuable insight into emissions of N_2_O from these soils. The range of land‐use emission factors (EFs) for peatlands varies widely, largely based on the particular soil type and management. The default IPCC Tier 1 EF for drained cropland on temperate and boreal organic soils is 13 ± 5 kg N_2_O‐N ha^−1^ year^−1^ (IPCC 2014). The UK currently uses a Tier 2 (country‐specific) emission factor of 16.3 ± 9.3 kg N_2_O‐N ha^−1^ year^−1^ (Evans et al. 2023). In this study we see highly variable emissions between years, corresponding to different management activities which are comparable to both estimates. The mean monthly emission of 0.50 ± 0.17 kg N_2_O‐N ha^−1^ for all measurements equates to approximately 6.0 ± 2.0 kg N_2_O‐N ha^−1^ year^−1^. Excluding data from 2021 during the high emission period when the potato crop was present, the monthly emission from the fields is 0.26 ± 0.04 kg N_2_O‐N ha^−1^ and equates to approximately 3.1 ± 0.5 kg N_2_O‐N ha^−1^ year^−1^. Cumulative emissions over the 8‐month period of measurements in 2021 during the potato crop when water tables were high give an annual estimate of approximately 13.8 kg N_2_O‐N ha^−1^ year^−1^ if the missing four winter months are accounted for (11.8 kg N_2_O‐N ha^−1^, with an additional 4 months estimated at 0.5 kg N_2_O‐N ha^−1^ per month), which is similar in magnitude to the IPCC Tier 1 EF for drained cropland on organic soils, but still lower than the UK Tier 2 value. During the remainder of the measurements, measured annual emissions were much lower than either the IPCC Tier 1 or UK Tier 2 EFs.

The current UK inventory approach applies a single N_2_O EF for cropland on peat (16.3 ± 9.3 kg N_2_O‐N ha^−1^ year^−1^; Evans et al. 2023), which does not differentiate according to crop type, management or peat depth. The value which is based on observations from sixteen cropland sites in the UK (Taft et al. 2017) and in climatically similar (maritime temperate) areas of continental Europe (e.g., Petersen et al. 2012; Beyer and Höper 2015; Tiemeyer et al. 2016, 2020). These studies were all undertaken using flux chamber measurements and varied widely in N_2_O emissions, with the highest N_2_O emissions reported for spring barley and potato crops (38 and 61 kg N_2_O‐N ha^−1^ year^−1^, respectively) in Western Denmark (Petersen et al. 2012), and lower than expected emissions at a maize site receiving pig slurry (1.45 kg N_2_O‐N ha^−1^ year^−1^) in Northwest Germany (Beyer and Höper 2015). Peaks in N_2_O emissions were attributed to fluctuating groundwater levels and interactions with nitrate availability at depth and vegetation cover, but clear drivers were not always apparent.

It is worth noting that IPCC inventory methods require separate reporting of N_2_O emissions derived from N fertilisation (either synthetic or animal waste) and those derived from soil mineralisation. In practice these can be difficult to distinguish in field measurements, and as a result there is some risk that both the IPCC Tier 1 and UK Tier 2 EFs for N_2_O emissions from agricultural soils could incorporate fertiliser‐derived fluxes, leading to possible double‐counting. The usual approach to avoid this issue is to run paired chamber measurements with and without fertiliser application, with N_2_O emissions in the unfertilised plots attributed to soil mineralisation, and any additional emissions from the fertilised plots attributed to fertiliser application (e.g., Cowan et al. 2019). However, our continuous data over several crop rotations call this approach into question, at least for cultivated lowland peat soils. Contrary to what would be expected, application of N fertiliser had no immediate measurable impact on N_2_O emissions from the soils in this study. While emissions after fertiliser application are not always sizable (and more often than not fall below the 1% threshold used by the IPCC (Cowan et al. 2020)), the lack of a response within the first 30 days after application for any of the four applications of mineral nitrogen could be considered surprising given the environmental conditions (e.g., temperate, damp, carbon‐rich soils). On the other hand, these results are consistent with those of long‐term N fertilisation experiments and monitoring studies of organic‐rich semi‐natural soils, which show progressive (‘chronic’) enrichment of the large organic matter pool, eventually leading to an excess of mineral N in the ecosystem, and subsequently to loss of excess N as both gaseous N_2_O and dissolved nitrate (e.g., Lovett and Goodale 2011; Oulehle et al. 2021). As resources available did not allow for the inclusion of soil nitrate measurements, we cannot include this in our analysis but would recommend future studies attempt to monitor nitrate concentrations in agricultural peatland soils when possible, especially at different depths. This contrasts with the ‘acute’ effects of short‐term N fertilisation of mineral soils, whereby the internal N cycle is (in the absence of high rates of plant uptake) rapidly overwhelmed by the capacity of the soil to assimilate N, leading to immediate N_2_O emissions. It is also likely that, in well‐managed agricultural systems, the amount and timing of N fertiliser applications will be adjusted to meet but not exceed plant N demand, thus minimising the conversion of excess N to N_2_O. In light of these observations, we consider that it may be inappropriate (and arguably meaningless) to seek to separate ‘fertiliser’ and ‘soil’ N_2_O when reporting emissions from peat or other organic‐rich soils, because both fertiliser and soil organic matter mineralisation lead to enrichment of a single mineral N pool from which all N_2_O emissions derive. Several previous studies of N_2_O emissions from cultivated peat soils also suggest little or no immediate response to N fertiliser application (e.g., Regina et al. 2004; Petersen et al. 2012), suggesting that our results are not unusual in this respect.

Apart from fertilisation, agricultural activities had variable impacts on N_2_O emissions. When comparing the impact of tillage events between years, it was only after the tillage event in October of 2023 that sizable N_2_O emissions were observed with a release of approximately 0.5 kg N_2_O‐N ha^−1^ (over a period of 4 weeks after tillage). This is the equivalent of a 50 kg N ha^−1^ fertiliser application using the IPCC default EF of 1%. Emissions of N_2_O after tillage events are common and attributed to mineralisation of incorporated organic N in crop residues, the magnitude of which is sometimes akin to an application of N fertiliser (Merbold et al. 2014; Cowan et al. 2016). There was a peak in emissions of N_2_O in the summer of 2023, midway through the growth of the bean crop, although this does not appear to be the result of environmental changes. As a legume crop, the beans would have been contributing to biological nitrogen fixation in the soil, which can produce N_2_O emissions (e.g., Hidalgo‐García et al. 2023); however, emissions associated with legume growth are often considered to be negligible as most fixed nitrogen is fixed by the plant (Li et al. 2021). The consistent lack of response to the addition of nitrogen fertilisers may indicate that the peatland soil is already relatively high in available nitrogen and that the controlling factors in the microbial production of N_2_O are environmental rather than driven by surface activity, which is similar to the Tier 2 EF method applied for UK soils as outlined by Evans et al. (2023).

Emissions of N_2_O at the field site appear to be strongly curtailed by two thresholds in environmental conditions in the peat soils. Where thresholds in both soil temperature (approximately 12°C) and VWC (approximately 65%) are exceeded and coincide, this results in an exponential increase in N_2_O emissions at the field site with further temperature increases. The representativeness of the soil conditions measured by the probes may vary when comparing against soils within the flux footprint at times. There will inevitably be an unknown element of spatial variability in soil moisture and temperature across the fields as well as the impact of upkeep activities within the tower enclosure (e.g., weed growth and shading which was more intense in the 22/23 period). Regardless, our data provide evidence of a strong link between these soil properties and N_2_O flux at the site. While many studies have focused on conditions in the surface soils (top 15 cm) to determine drivers for N_2_O emissions, the peatland soils at this field site respond to conditions in at least the top 60 cm of soil, potentially deeper (depending on the peat depth and water table management). This is consistent with flux tower data from the UK and elsewhere (e.g., Evans et al. 2023) showing a positive relationship between CO_2_ emissions and water table depth, which suggests that organic matter is being mineralised throughout the full depth of aerated peat.

There are numerous reasons that these thresholds could be of high importance to the processes that produce N_2_O in soils. N_2_O is primarily the by‐product of denitrification in soils in which nitrate (${\mathrm{NO}}_{3}^{-}$) is converted to inert nitrogen gas (N_2_). Microbial activity is strongly correlated to temperature and other studies have reported similar threshold behaviours, but at a variety of temperatures in different environments (e.g., Drewer et al. 2011; Gu et al. 2020). The difference in soil temperatures between the two tillage events in 2023 may explain why no N_2_O emissions were observed during one event (mean surface temperature of 7°C in March 2023), while emissions from another similar event were immediate and significantly larger (mean surface temperature of 14°C in October 2023).

Denitrification rates and also the ratio of nitrogen that is released as N_2_O are highly dependent on the anaerobicity of soils (e.g., Davidson et al. 2000). Conditions in which oxygen is limited (e.g., very wet soils with no exchange with the atmosphere and thus no oxygen exposure) will favour denitrification; however, N_2_O is often consumed when soils are highly anaerobic (all N_2_O is converted to N_2_), and full peat re‐wetting therefore results in low or zero N_2_O emissions (Liu et al. 2020). A WFPS of approximately 60% has been identified as the point at which N_2_O production is maximised by denitrification processes, although this may vary by soil type and conditions (Robertson and Groffman 2015). In comparison to mineral soils, peat soils become highly porous when dry, thus airflow can reach lower in the soil profile, and gaseous emissions can escape from lower in the profile than from soils such as grasslands or arable fields where clay content and soil compaction prevent air flow. Lateral N_2_O losses via subsurface drains and the ditch network are also likely (e.g., Silverthorn et al. 2025) and may have contributed to N_2_O emissions measured at the flux tower. Peatland soils can shrink when the water table drops and can stay saturated for longer (Barber et al. 2004), but the peat profile was never waterlogged at our study site, so it is unlikely that anaerobic conditions ever restricted N_2_O emissions.

In this study, we clearly identify a lasting period of exceptionally high N_2_O emissions during 2021. These emissions appear to derive predominantly from that part of the soil in which the key thresholds of temperature and soil anaerobicity (soil moisture) are reached to allow for an area of high denitrification potential in the soil profile (at approximately 50 to 60 cm depth). While it is clear that these high emissions were associated with a specific combination of environmental conditions, it also appears that these conditions were to a large extent the result of field management factors associated with the cultivation of the potato crop. Specifically, the crop was irrigated, and water levels were maintained at a relatively high level, leading to the high soil moisture level at 50–60 cm depth. The crop also received relatively higher rates of N fertilisation (104 kg N ha^−1^ over the cropping period). Surface soil temperatures in 2021 were markedly higher than in subsequent years (despite the 2022 heatwave) which we attribute to solar heating of the exposed and ridged soil surface under the potato crop, a pattern also observed by Kandel et al. (2018). These higher temperatures likely contributed to accelerated nitrification and denitrification compared to subsequent years, where surface shading by more productive wheat and bean crops led to lower soil surface temperatures. Previous chamber‐based studies on drained peatlands in Denmark also showed higher N_2_O emissions from potato versus cereal crops (Petersen et al. 2012; Kandel et al. 2018), although in the latter case the highest emissions occurred outside of the cropping period. Regina et al. (2004) did not observe higher emissions from a field in Southern Finland under a potato crop compared to grass or barley (and emissions from a fallow year were higher), but again they reported higher emissions outside of the cropping period, in their case associated with thaw events.

While it would be unwise to conclude too much based on 3 years of data from a single site, the extent to which we have been able to relate N_2_O emissions to environmental variables linked to crop management does suggest that these emissions can be expected to vary as a function of agricultural practices. Specifically, crops requiring high rates of fertiliser and irrigation (such as root and salad crops) might be expected to have higher associated N_2_O emissions, as will crops that expose the soil to surface heating (in our case this was most marked for potatoes). Conversely, more productive crops such as cereals that have higher N demand, lower water demand and which provide greater shading of the soil surface may result in lower N_2_O emissions. Given the large (three to four‐fold) differences in annual emissions observed between years with different cropping at our study site, these results—if borne out by further studies—may justify the application of different N_2_O emission factors for different crops (e.g., vegetables vs. cereals) grown on peat.

The control of water table depths in peatlands has been linked to the emission of CO_2_ and CH_4_, and it has been suggested in previous studies that changing water table depths of peatland agriculture can reduce these emissions (Emissions of CO_2_ from this field site will be reported separately as part of a further analysis of carbon stocks at the site, Figure S6). A global meta‐analysis by Evans et al. (2023) suggested that halving the water table depth in all drained agricultural peatlands could reduce emissions by the equivalent of over 1% of global anthropogenic emissions, although the impact on N_2_O is not included in that analysis. While fluxes of N_2_O in this study did not correlate directly with water table depth, the influence of higher water tables can be seen in the high moisture content and resulting high N_2_O fluxes observed in 2021. Although it is not possible to entirely separate the influence of soil moisture from other factors such as fertilisation rates and soil temperature, as discussed above, this study does suggest that changing the water table in agricultural peatlands could have significant impacts on N_2_O emissions, with higher water levels resulting in additional emissions of around 10 kg N_2_O‐N ha^−1^ year^−1^ (4 t CO_2eq_ ha^−1^ year^−1^ over a 100‐year period) compared to years when water levels were managed at lower levels. This finding seemingly creates some conflict with measures to reduce CO_2_ emissions by raising water levels, although this will not necessarily always be the case, for example in the case of crops with high N demand, or if water levels are raised sufficiently to restrict both CO_2_ and N_2_O production. Furthermore, the study only included a single field site with a single year of high‐water table flux measurements, so it would be premature to assume the N_2_O emissions in this study would be replicated at larger scales. One valuable outcome of this study is that we may be able to explain why we do see such a wide range of emission factors reported for peatland soils in previous studies, particularly those with managed drainage for agriculture. It is rare for studies to capture long‐term temporal spatially integrated N_2_O fluxes from agricultural soils, and to clearly identify the underlying drivers of microbial activity within the soil profile. Based on the observations made in this study, we would recommend future studies on peatland fluxes of N_2_O take efforts to monitor soil conditions down to at least 1 m in the soil profile and consider the influence that water table, crop type and other management activities may have on surface fluxes.

## 5 Conclusions

Our study shows that emissions of N_2_O from farmed peatland soils are highly variable and strongly dependent on environmental variables. We observe an exponential increase in the variability of measured N_2_O fluxes at our peatland field site when soil temperature increases beyond 12°C and coincides with a volumetric water content that exceeds 65% at depths up to 1 m below the soil surface. Water table depth in the fields impacted the moisture content of the soil in the 0.5 to 1.0 m depth, which appears to have drastically impacted emissions at the site, which ranged from 3 to 13 kg N_2_O‐N ha^−1^ year^−1^. Our results suggest that the nitrogen dynamics of the peatland soils vary considerably in comparison to mineral soils, and that conventional N_2_O accounting methods that split land type and fertiliser application contributions to emissions (primarily developed and used on mineral soils) are not suitable in determining emission factors for agricultural peatlands. The lack of immediate N_2_O emission after nitrogen application and high temporal variability and strong response to environmental conditions within the peat soils suggest that any attempt to categorise emission factors at the field scale requires high temporal, long‐term measurements, such as those achieved by eddy covariance or an auto‐chamber setup. These factors also suggest that historic experiments carried out using manual chambers are susceptible to very large uncertainties or systematic bias as a result of poor interpolation practices (e.g., there is no accurate process‐based model to gap‐fill N_2_O flux). While one field study is not enough to fully inform farmers of best practice regarding N_2_O mitigation, we highlight the need for a better understanding of nitrogen availability in peatland soils. Our data suggest that nitrogen application to agricultural peatland risks exceeding plant requirements as soils are not nitrogen limited in the same way as mineral soils. Farmers with peatland soils may wish to trial fractionally lower nitrogen applications to reduce costs and environmental burden. Different crops also appear to lead to markedly different N_2_O emissions. To establish a better understanding of the high variability in N_2_O fluxes observed from farmed peatland soils and improve inventory estimates of N_2_O, we recommend that further studies investigate the impact of water table controls and the effects of soil moisture down to 1 m in the soil, which may be the primary cause of the wide variability in reported emission factors for peatland soils. We also recommend further investigation of the full GHG balance of water table alterations (CO_2_, CH_4_ and N_2_O) in peatland soils so that farmers can be given accurate advice for best practice in terms of climate impact of crop management.

## Author Contributions

**Nicholas Cowan:** data curation, formal analysis, investigation, methodology, visualization, writing – original draft. **Alex Cumming:** data curation, investigation, methodology, writing – review and editing. **Ross Morrison:** conceptualization, funding acquisition, investigation, methodology, resources, supervision, validation, visualization, writing – review and editing. **Hannah Clilverd:** investigation, writing – original draft, writing – review and editing. **Luke Palmer:** conceptualization, investigation, methodology, project administration, resources, writing – review and editing. **Chris D. Evans:** conceptualization, funding acquisition, investigation, project administration, resources, supervision, writing – original draft, writing – review and editing.

## Funding

This work was supported by the Department for Energy Security and Net‐Zero.

## Conflicts of Interest

The authors declare no conflicts of interest.

## Supporting information


**Figure S1:** (a) Daily cumulative rainfall, (b) daily mean volumetric water content (VWC), (c) daily mean temperature of the soil (both at top 10 cm) and (d) water table depth at the Stowbridge Farm flux tower site from 2022 to 2024.
**Figure S2:** Time series of half‐hourly measured air and soil temperature at the Stowbridge Farm flux tower site from 2021 to 2024.
**Figure S3:** (a) Wind rose of all N_2_O flux measurements that passed quality control, showing the frequency of wind speeds by direction. (b) Wind rose of the same dataset, indicating the estimated distance from the flux mast at which the peak flux contribution occurred.
**Figure S4:** Fluxes of N_2_O from 2021 to 2024 plotted against the maximum recorded temperature in the soil profile (5–100 cm), grouped in colour by the maximum recorded VWC in the soil profile (5–100 cm). Data points represent fluxes and data measured over 30 min. Fit line represents the formula *y* = exp (*x*).
**Figure S5:** Fluxes of N_2_O from 2021 to 2024 plotted against the water table level in the soil, with a colour scale of the maximum recorded VWC in the soil profile (5–100 cm). Data points represent fluxes and data measured over 30 min.
**Figure S6:** Diurnal fluxes of CO_2_ are separated seasonally and by year. Fluxes are binned by the crop that was present in the field during flux measurements. Boxplots represent the median and 25th and 75th percentiles of flux data, respectively (whiskers represent the 95th percentiles).

## Data Availability

Data supporting this study is openly available from Zenodo.org at https://doi.org/10.5281/zenodo.17513191.
