# Supplementary material for: Measuring Fluxes of Nitrous Oxide (N2O) From an Intensively Farmed Wasted Peatland Field in the UK Using the Eddy Covariance Method
Source: Glob Chang Biol. 2025 Nov 21;31(11):e70619. doi: 10.1111/gcb.70619 (PMC12639271; doi:10.1111/gcb.70619)
Supplement: Supplementary file 1 — Figure S1: (a) Daily cumulative rainfall, (b) daily mean volumetric water content (VWC), (c) daily mean temperature of the soil (both at top 10 cm) and (d) water table depth at the Stowbridge Farm flux tower site from 2022 to 2024. Figure S2: Time series of half‐hourly measured air and soil temperature at the Stowbridge Farm flux tower site from 2021 to 2024. Figure S3: (a) Wind rose of all N2O flux measurements that passed quality control, showing the frequency of wind speeds by direction. (b) Wind rose of the same dataset, indicating the estimated distance from the flux mast at which the peak flux contribution occurred. Figure S4: Fluxes of N2O from 2021 to 2024 plotted against the maximum recorded temperature in the soil profile (5–100 cm), grouped in colour by the maximum recorded VWC in the soil profile (5–100 cm). Data points represent fluxes and data measured over 30 min. Fit line represents the formula y = exp (x). Figure S5: Fluxes of N2O from 2021 to 2024 plotted against the water table level in the soil, with a colour scale of the maximum recorded VWC in the soil profile (5–100 cm). Data points represent fluxes and data measured over 30 min. Figure S6: Diurnal fluxes of CO2 are separated seasonally and by year. Fluxes are binned by the crop that was present in the field during flux measurements. Boxplots represent the median and 25th and 75th percentiles of flux data, respectively (whiskers represent the 95th percentiles). [file GCB-31-e70619-s001.docx]

**Measuring fluxes of nitrous oxide (N_2_O) from an intensively farmed wasted peatland field in the UK using the eddy covariance method**

# Supplementary Materials


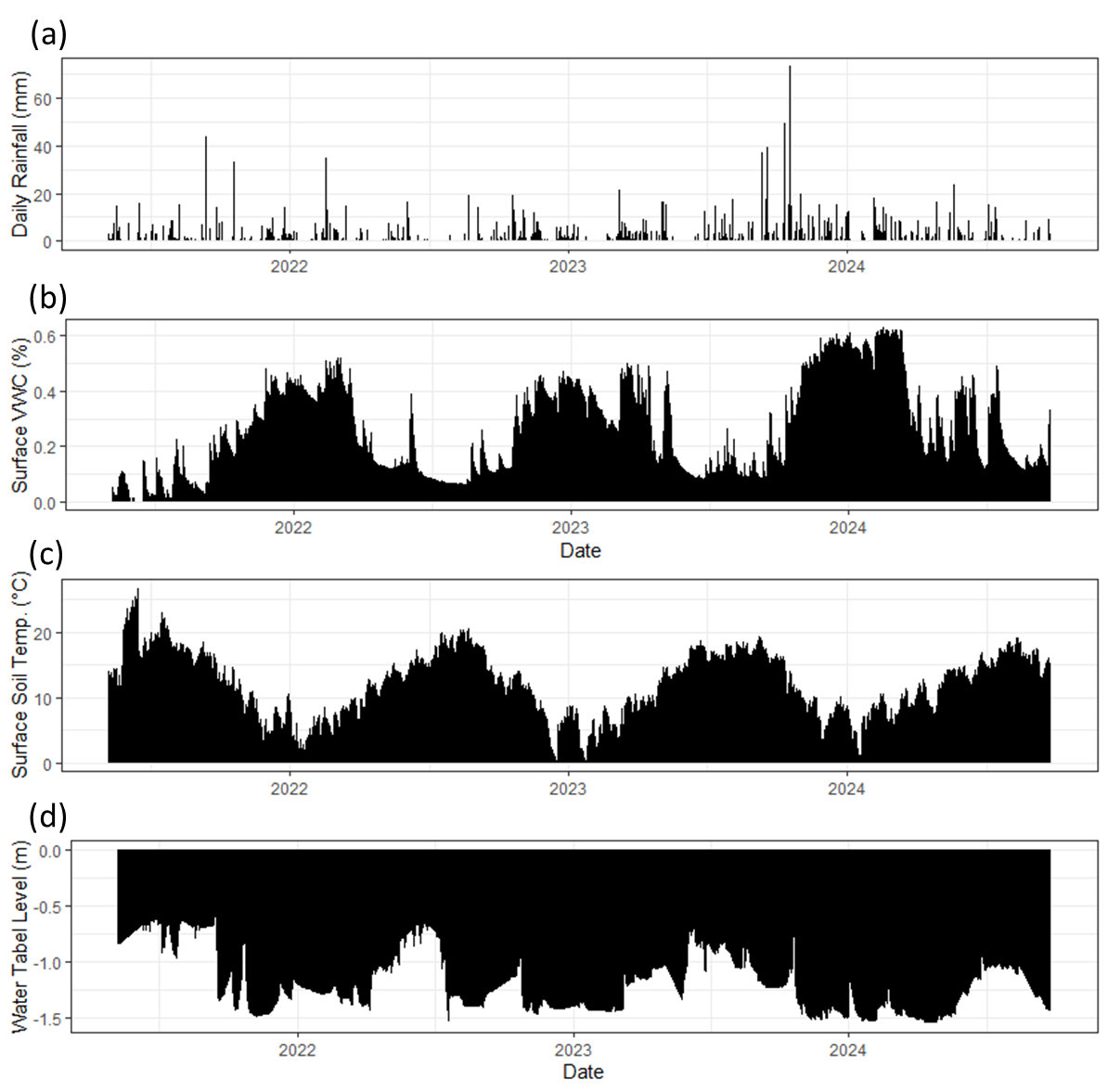


**Figure S1** (a) Daily cumulative rainfall, (b) daily mean volumetric water content (VWC), (c) daily mean temperature of the soil (both at top 10 cm) and (d) water table depth at the Stowbridge Farm flux tower site from 2022 to 2024.


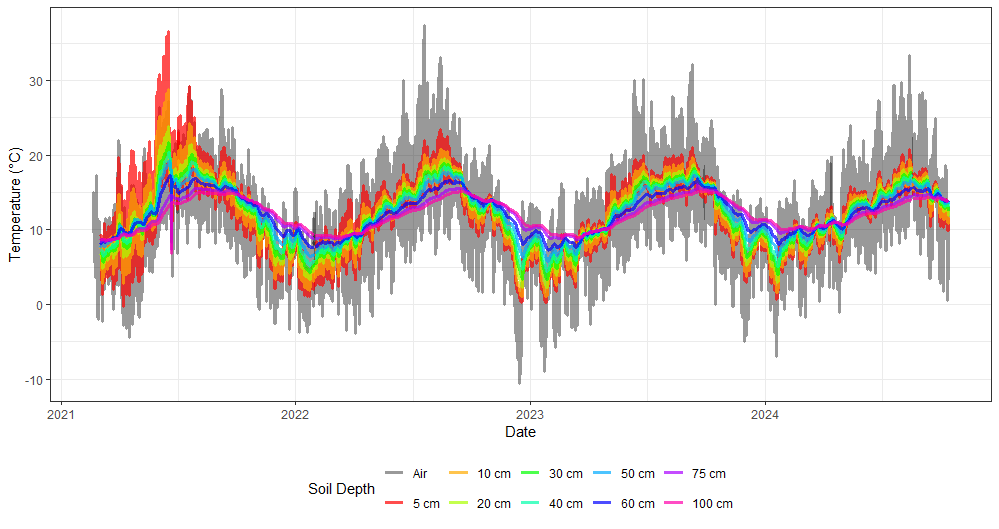


**Figure S2**. Time series of half-hourly measured air and soil temperature at the Stowbridge Farm flux tower site from 2021 to 2024.


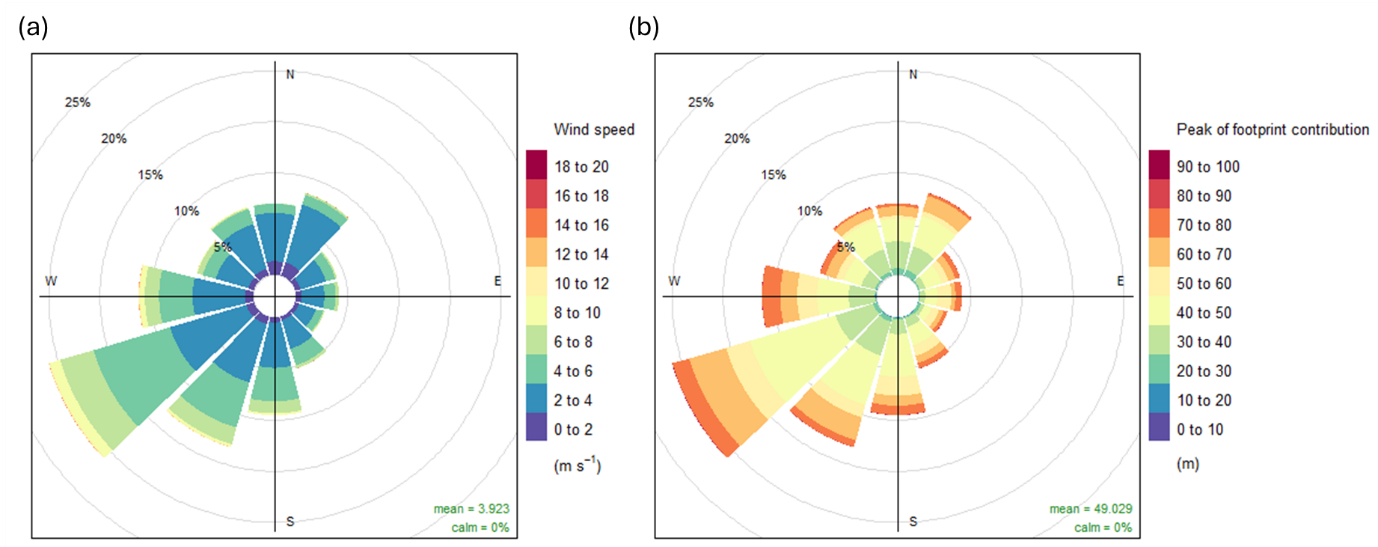


**Figure S3** (a) Wind rose of all N_2_O flux measurements that passed quality control, showing the frequency of wind speeds by direction. (b) Wind rose of the same dataset, indicating the estimated distance from the flux mast at which the peak flux contribution occurred.


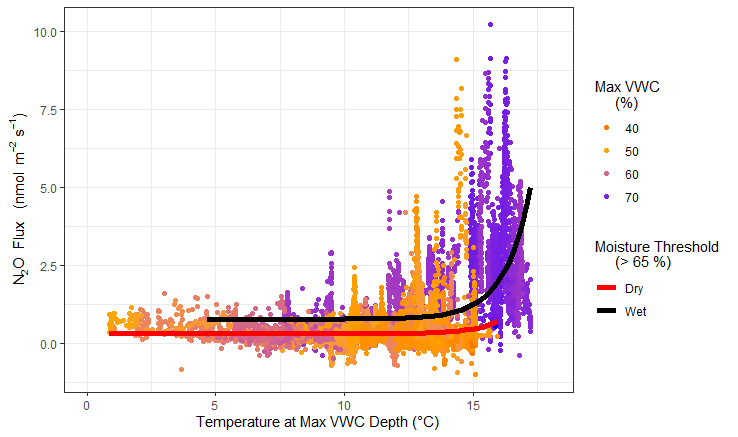


**Figure S4** Fluxes of N_2_O from 2021 to 2024 plotted against the maximum recorded temperature in the soil profile (5-100 cm), grouped in colour by the maximum recorded VWC in the soil profile (5-100 cm). Data points represent fluxes and data measured over 30 minutes. Fit line represents the formula y = exp(x).


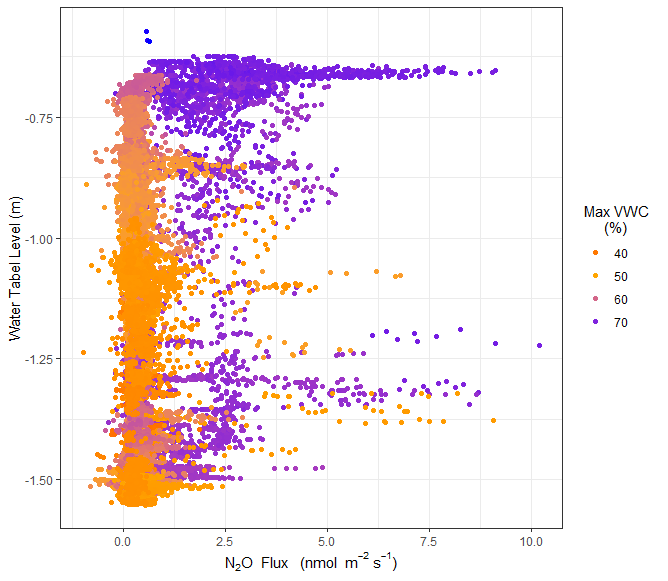


**Figure S5** Fluxes of N_2_O from 2021 to 2024 plotted against the water table level in the soil, with a colour scale of the maximum recorded VWC in the soil profile (5-100 cm). Data points represent fluxes and data measured over 30 minutes.


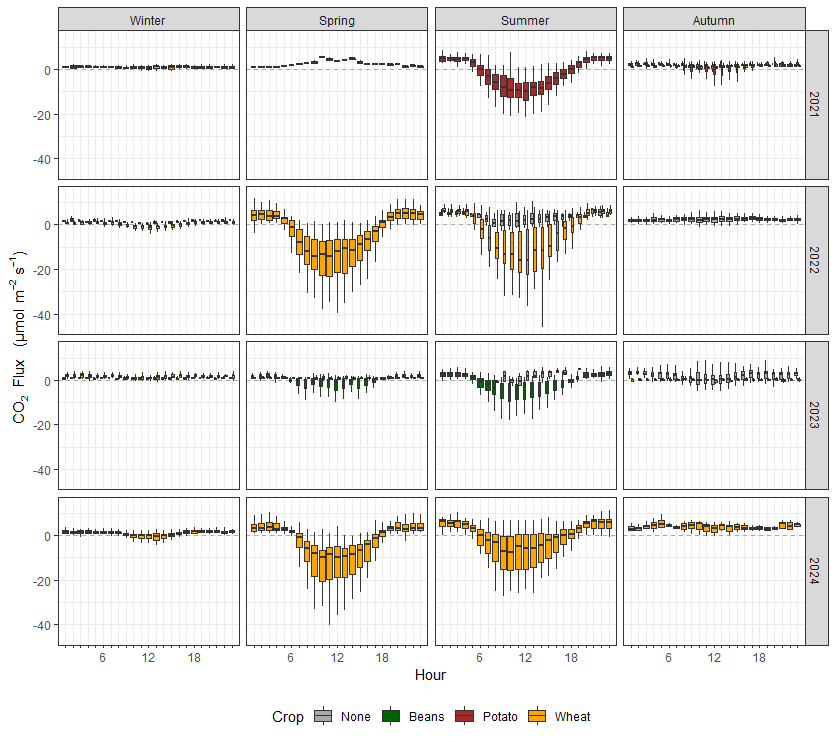


**Figure S6** Diurnal fluxes of CO_2_ are separated seasonally and by year. Fluxes are binned by the crop that was present in the field during flux measurements. Boxplots represent the median and 25th and 75th percentiles of flux data, respectively (whiskers represent the 95^th^ percentiles).
